# Quantitative group testing-based overlapping pool sequencing to identify rare variant carriers

**DOI:** 10.1186/1471-2105-15-195

**Published:** 2014-06-17

**Authors:** Chang-Chang Cao, Cheng Li, Xiao Sun

**Affiliations:** 1 State Key Laboratory of Bioelectronics, School of Biological Science and Medical Engineering, Southeast University, Nanjing, China

**Keywords:** Quantitative group testing, Random *k*-set pool design, Overlapping pool sequencing, Rare variants

## Abstract

**Background:**

Genome-wide association studies have revealed that rare variants are responsible for a large portion of the heritability of some complex human diseases. This highlights the increasing importance of detecting and screening for rare variants. Although the massively parallel sequencing technologies have greatly reduced the cost of DNA sequencing, the identification of rare variant carriers by large-scale re-sequencing remains prohibitively expensive because of the huge challenge of constructing libraries for thousands of samples. Recently, several studies have reported that techniques from group testing theory and compressed sensing could help identify rare variant carriers in large-scale samples with few pooled sequencing experiments and a dramatically reduced cost.

**Results:**

Based on quantitative group testing, we propose an efficient overlapping pool sequencing strategy that allows the efficient recovery of variant carriers in numerous individuals with much lower costs than conventional methods. We used random *k*-set pool designs to mix samples, and optimized the design parameters according to an indicative probability. Based on a mathematical model of sequencing depth distribution, an optimal threshold was selected to declare a pool positive or negative. Then, using the quantitative information contained in the sequencing results, we designed a heuristic Bayesian probability decoding algorithm to identify variant carriers. Finally, we conducted *in silico* experiments to find variant carriers among 200 simulated *Escherichia coli* strains. With the simulated pools and publicly available Illumina sequencing data, our method correctly identified the variant carriers for 91.5–97.9% variants with the variant frequency ranging from 0.5 to 1.5%.

**Conclusions:**

Using the number of reads, variant carriers could be identified precisely even though samples were randomly selected and pooled. Our method performed better than the published DNA Sudoku design and compressed sequencing, especially in reducing the required data throughput and cost.

## Background

Rare variants are responsible for a large portion of the heritability of some common complex human diseases [1,2]. Genome-wide association studies have focused on the contribution of variants of low minor allele frequency (MAF 0.5–5%), or of rare variants (MAF < 0.5%) [2]. The functional and evolutionary impacts of rare variants have been reported; therefore, large-scale screening for disease-associated rare variants is becoming increasingly important [3,4]. One major application of rare variants genotyping is in screens for rare genetic disorders such as Tay–Sachs disease and thalassemia [5].

Because of the extremely low frequency of rare variants, sample sizes must be large enough to guarantee efficient observations. The cost of DNA sequencing has dropped dramatically with the introduction of the massively parallel sequencing technologies. However, identifying rare variant carriers by sequencing individual samples separately remains prohibitively expensive because of the huge challenge of constructing sequencing libraries for thousands of samples [6,7]. Barcoding has been developed as a powerful approach to cost-effectively determine the genotype of each individual [7]. To further reduce the cost of large-scale screens for rare variant carriers, several techniques based on the group testing theory [8] and compressed sensing [9,10] to construct overlapping pool sequencing strategies have been used. These strategies have helped decrease the sequencing times for rare variant carrier identification and further lower the cost [11-14].

Because a large number of samples are pooled together and then sequenced, overlapping pool sequencing uses fewer pools to identify rare variant carriers among large numbers of individuals. Thus, overlapping pool sequencing can vastly reduce the time and cost for DNA library preparation. Some overlapping pool sequencing programs return true/false values after testing each pool; this scheme was adopted by Erlich et al. [11], Prabhu and Pe’Er [12], and Cao et al. [14], who used the number of normal and variant reads in each pool to determine whether a pool contained carriers. However, the quantitative information in the sequencing data, which can be used to estimate the percentage of variant chromosomes in a pool, is missed in these methods. Quantitative group testing is an alternative scheme that takes the quantitative information into account, thus rare variant carriers can be identified efficiently [13].

We propose an efficient random overlapping pool sequencing strategy with quantitative group testing for the identification of rare variant carriers using massively parallel sequencing data. Because of the excellent performance of random designs in classic group testing [15,16], we employed a random *k*-set pool design [17] to mix samples. The parameters of the random *k*-set pool design can be selected appropriately according to an indicative probability value. Based on a depth model for pooled sequencing, we calculated the optimal cut-off of the number of reads containing variants to distinguish pools containing variant carriers from those that do not. Using the quantitative information contained in the sequencing data, we designed a heuristic Bayesian decoding algorithm to identify variant carriers accurately. Compared with the DNA Sudoku algorithm [11] and compressed sequencing [13], our method required less data throughput. Finally, we applied our method to identify variant carriers among 200 simulated *Escherichia coli* strains using simulated pools and publicly available Illumina sequencing data. The results showed that our method could successfully identify carriers for 91.5–97.9% of the variants with frequencies ranging from 0.5 to 1.5%.

## Methods

### Random *k*-set pool design

Random *k*-set pool designs were first proposed by Bruno et al. [15] for efficient DNA clone library screening. In such a design, each clone is pooled in exact *k* pools that are chosen with equal chance. Random *k*-set pool designs are easy to specify for any number of pools and are known to be efficient in classic group testing, but they have not been used in real situations, partly because of the presence of different sample sets with identical test sets that are indistinguishable when the test results are qualitative [16]. However, this problem can be overcome by quantitative tests such as sequencing experiments.

For *n* samples containing *d* positive samples, the basic objective of a random *k*-set pool design is to identify all the positive samples with a small number of pooled tests. In such a design, each sample occurs in exact *k* pools, and a pool is defined as positive only when it contains at least one positive sample; otherwise, it is defined as negative. For a random *k*-set pool design with *t* pools, Hwang [17] calculated the probability that a given set of *i* pools is a negative one (Eq. (1)) and the expected number of negative pools (Eq. (2).

(1) $$\begin{array}{l} \mathrm{NEG}\left(i\right)=\sum_{h=i}^{t}{\left(-1\right)}^{h-i}\left(\begin{array}{c} t-i\\ h-i \end{array}\right)\left(\begin{array}{c} \left(\begin{array}{c} t-h\\ k \end{array}\right)\\ d \end{array}\right)/\left(\begin{array}{c} \left(\begin{array}{c} t\\ k \end{array}\right)\\ d \end{array}\right)\\ \;\times i\in \left[n\_\min,n\_\max\right] \end{array}$$

(2) $$E=\sum_{i=n\_\min}^{n\_\max}i\left(\begin{array}{c} t\\ i \end{array}\right)\mathrm{NEG}\left(i\right)$$

where *n_min* and *n_max* are the minimum and maximum number of negative pools, respectively, and *h* is a temporary variable. *n_max* = *t - k*, and *n_min* = 0 (if *dk* ≥ *t*) or *t - dk* (if *dk* < *t*).

To evaluate the performance of random designs, Barillot et al. [18] proposed that the number of unresolved negative samples ($\bar{N}$) can be taken as a criterion. An unresolved negative sample is defined as a negative sample that occurred only in positive pools, as a result, its status can only be confirmed by testing it separately. Negative samples that are contained in at least one negative pool can confidently be determined as negative; therefore, Hwang [17] calculated the expectation (Eq. (3)) and probability distributions (Eq. (4)) for the number of unresolved negative samples.

(3) $$E\left(\bar{N}\right)=\left(n-d\right)\sum_{i=n\_\min}^{n\_\max}\left(\begin{array}{c} t\\ i \end{array}\right)\mathrm{NEG}\left(i\right)\left[\left(\begin{array}{c} t-i\\ k \end{array}\right)-d\right]/\left[\left(\begin{array}{c} t\\ k \end{array}\right)-d\right]$$

(4) $$\begin{array}{l} P\left(\bar{N}=j\right)=\sum_{i=n\_\min}^{n\_\max}\left(\begin{array}{c} t\\ i \end{array}\right)\mathrm{NEG}\left(i\right)\left(\begin{array}{c} \left(\begin{array}{c} t-i\\ k \end{array}\right)-d\\ j \end{array}\right)\\ \;\times (\left(\begin{array}{c} \left(\begin{array}{c} t\\ k \end{array}\right)-\left(\begin{array}{c} t-i\\ k \end{array}\right)\\ n-d-j \end{array}\right)/\left(\begin{array}{c} \left(\begin{array}{c} t\\ k \end{array}\right)-d\\ n-d \end{array}\right) \end{array}$$

For quantitative group testing, negative pools are used to recognize the negative samples and the test results of positive pools are used to distinguish real positive samples from unresolved negative samples. When the number of positive pools is less than the sum of unresolved negative samples and positive samples, numerous solutions are possible. Intuitively, a design that has more positive pools and fewer unresolved negative samples will have a higher probability of identifying all the positive samples correctly. Therefore, we designed an indicative probability (*PI*; Eq. (5)) to evaluate the performance of random *k*-set designs in quantitative group testing. *PI* is the probability that positive pools are more than the sum of unresolved negative samples and real positive samples; therefore, designs with high *PI*s will perform better than designs with low *PI*s.

(5) $$\mathrm{PI}=\sum_{i=p\_\min}^{p\_\max}\left(\begin{array}{c} t\\ t-i \end{array}\right)\mathrm{NEG}\left(t-i\right)\sum_{j=0}^{i-d}P\left(\bar{N}=j\right)$$

where *p_min* and *p_max* are the minimum and maximum number of positive pools, respectively, *p_min* = *t* - *n_max*, and *p_max* = *t* - *n_min*. The derivation of Eq. (5) is given in Appendix 1.

### Optimal cut-off value for pooled sequencing

For overlapping pool sequencing, the DNA samples are mixed and then sequenced. Samples from variant carriers are treated as positive and samples from normal individuals are treated as negative. To distinguish positive pools containing variant carriers from negative pools consisting of normal individuals, the cut-off for the number of reads containing variants must be selected carefully to declare whether a pool contains carriers or not. Ideally, the cut-off value must guarantee that the minimum error rates are obtained, including false-positive and false-negative rates.

The variation of sequencing depth distribution is significantly greater than the mean [19,20]; therefore, in recent studies, negative binomial distribution rather than Poisson distribution has been used to model sequencing depth. Following the study reported by Miller et al. [21], we used a negative binomial model to estimate the sequencing depth distribution. Given the average sequencing depth *D*, the depth that represents the number of times a nucleotide is read follows a negative binomial distribution $\mathrm{NB}\left(\frac{D}{r-1},\frac{1}{r}\right)$ where *r* is the variance/mean ratio; *r* is related to sequencing platforms and genomes and can be estimated from sequencing results (Eq. (6)).

(6) $$P\left(\mathrm{depth}=i\right)=\mathrm{NB}\left(i;\frac{D}{r-1},\frac{1}{r}\right)$$

For a pool with *N* samples, given sequencing depth *D* and average sequencing error rate *p*_*error*_, the probabilities *P*_*nv*_(*N*_*v*_) and *P*_*pv*_(*N*_*v*_) that *N*_*v*_ reads containing variants are observed in negative pools and positive pools, respectively, are given by Eqs. (7) and (8). For a locus sequenced *i* times, the number of sequencing errors follows a binomial distribution *Bin*(*i*, *p*_*error*_).

(7) $$P_{\mathrm{nv}}\left(N_{v}\right)=\sum_{i=N_{v}}^{\infty }\mathrm{NB}\left(i;\frac{D}{r-1},\frac{1}{r}\right)\mathrm{Bin}\left(N_{v};i,p_{\mathrm{error}}\right)$$

(8) $$P_{\mathrm{pv}}\left(N_{v}\right)=\sum_{x=0}^{N_{v}}\left\{\begin{array}{c} \sum_{i=x}^{\infty }\mathrm{NB}(i;\frac{\left(1-p\right)D}{r-1},\frac{1}{r})\mathrm{Bin}\left(x;i,p_{\mathrm{error}}\right)\times \\ \sum_{j=N_{v}-x}^{\infty }\mathrm{NB}(j;\frac{\mathrm{pD}}{r-1},\frac{1}{r})\mathrm{Bin}\left(j-N_{v}+x;j,p_{\mathrm{error}}\right) \end{array}\right\}$$

Similarly, the probabilities *P*_*nn*_(*N*_*n*_) and *P*_*pn*_(*N*_*n*_) that *N*_*n*_ reads without variants are observed in negative pools and positive pools, respectively, are given by Eqs. (9) and (10).

(9) $$P_{\mathrm{nn}}\left(N_{n}\right)=\sum_{i=N_{n}}^{\infty }\mathrm{NB}\left(i;\frac{D}{r-1},\frac{1}{r}\right)\mathrm{Bin}\left(i-N_{n};i,p_{\mathrm{error}}\right)$$

(10) $$P_{\mathrm{pn}}\left(N_{n}\right)=\sum_{x=0}^{N_{n}}\left\{\begin{array}{c} \sum_{i=x}^{\infty }\mathrm{NB}(i;\frac{\left(1-p\right)D}{r-1},\frac{1}{r})\mathrm{Bin}\left(i-x;i,p_{\mathrm{error}}\right)\times \\ \sum_{j=N_{n}-x}^{\infty }\mathrm{NB}(j;\frac{\mathrm{pD}}{r-1},\frac{1}{r})\mathrm{Bin}\left(N_{n}-x;j,p_{\mathrm{error}}\right) \end{array}\right\}$$

where *p* is the percentage of variant chromosomes in the pool. In a positive pool with *N* diploid DNA samples and *c* heterozygous variant carriers, ignoring the bias in mixing samples, the percentage of the variant chromosomes is *p* = *c*/(2 *N*), while for haploid samples *p* = *c*/*N*. The derivations of Eqs. (7)–(10) are given in Appendix 1.

Given *P*_*nv*_(*N*_*v*_) and *P*_*pv*_(*N*_*v*_), the formula for the false-positive rate *F*_*p*_ and false-negative rate *F*_*n*_ in classifying pools with a cut-off value *T* can be constructed (Eqs. (11) and (12)).

(11) $$F_{p}=\sum_{i=T}^{\infty }P_{\mathrm{nv}}\left(i\right)$$

(12) $$F_{n}=\sum_{i=0}^{T-1}P_{\mathrm{pv}}\left(i\right)$$

The optimal cut-off *T* can be defined as the value that minimizes the maximum values of *F*_*n*_ and *F*_*p*_.

$$T=\arg\min\;\left\{\max\left(\mathrm{Fn},\mathrm{Fp}\right)|T\in \left[1,D\right]\right\}$$

### Decoding algorithm

Our decoding procedure involves two steps. In the first step, we identify negative pools according to the sequencing results and cut-off values for each pool. Samples that participate in any negative pools are classified as from normal individuals. Then, separate the real positive samples from unresolved negative samples according to the quantitative information in the sequencing results. The probability of observing the sequencing results under the exact set of variant carriers should be greater than taking other set of samples as variant carriers. Assuming *A* is the set of variant carriers, the probability that the sequencing result *O* is observed is given by Eq. (13).

(13) $$P\left(O|A\right)=\prod_{i=1}^{t}P\left(O_{\mathrm{iv}},O_{\mathrm{in}}|A\right)$$

where *O*_*iv*_ and *O*_*in*_ are the number of reads with and without variants in the *i*^th^ pool. Given that *A* is the set of variant carriers, if the *i*^th^ pool is negative, then *P*(*O*_*iv,*_*O*_*in*_*|A*) = *P*_*nv*_(*O*_*iv*_)*P*_*nn*_(*O*_*in*_); otherwise, *P*(*O*_*iv,*_*O*_*in*_*|A*) = *P*_*pv*_(*O*_*iv*_)*P*_*pn*_(*O*_*in*_).

For the second step, we designed a Bayesian decoding algorithm based on Eq. (13). To identify variant carriers among haploid samples, after excluding resolved negative samples, the rest of the samples form a set *A*_*0*_ = {*S*_*1*_,…,*S*_*c*_}. First, assuming that all the samples in *A*_*0*_ are variant carriers, we calculate the probability of observing the sequencing results and denote it as *Pmax_0*. Next, replace one positive sample in set *A*_*0*_ with a negative sample in turn and repeat the probability calculation. Denote the derived set that results in the maximum probability as *A*_*1*_ and the corresponding probability as *Pmax_1*. Replace *A*_*0*_ with *A*_*1*_ and repeat this step until *A*_*c*_ and *Pmax_c* are obtained. Finally, the set *A*_*i*_ that results in the maximum corresponding probability *Pmax_i* is defined as the set of variant carriers. These steps are written as Algorithm 1.

Two kinds of positive samples need to be considered while identifying variant carriers among diploid samples: heterozygous carriers and homozygous carriers. First, suppose that there are only heterozygous variant carriers; this is analogous to finding variant carriers among haploid samples. Then we present Algorithm 2 which is very similar to Algorithm 1 to recognize heterozygous and homozygous variant carriers.

Our decoding procedure to identify variant carriers among diploid samples is summarized in Algorithm 3. First, we suppose that only heterozygous variant carriers exist and run Algorithm 1 to find the set of variant carriers *C*_*0*_. Then, run Algorithm 2 to recognize heterozygous and homozygous variant carriers..

## Results and discussion

### Optimal cut-off value

**Figure 1:**
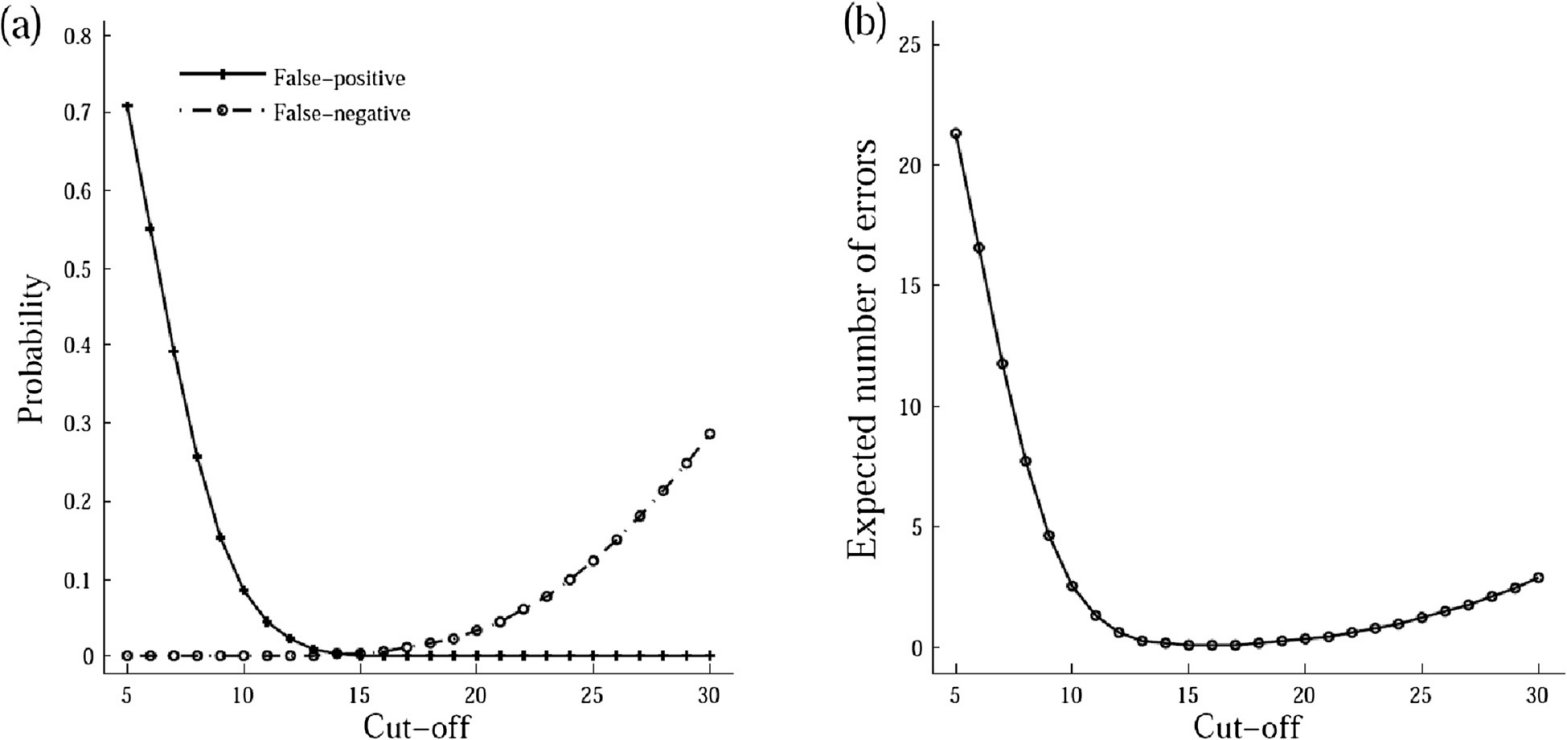
**Optimal cut-off values for pooled sequencing of 10 diploid samples. (a)** False-positive and false-negative probabilities for declaring whether a pool contains variant carriers. **(b)** Expected number of errors in classifying pools for an overlapping pool sequencing experiment with 30 negative pools and 10 positive pools.

To approximate the sequencing depth distribution for data obtained by using Illumina sequencing platforms, Miller et al. [21] found that the negative binomial distribution with the variance/mean ratio *r* of 3 performed much better than the Poisson distribution. Therefore, *r* was set to 3 in our simulation unless otherwise stated.For a pool consisting of 10 diploid samples, we calculated the false-positive and false-negative probabilities with different cut-off values when only one heterozygous variant carrier was allowed in the positive pool. The average sequencing error rate and whole depth were set to 0.01 and 600×, respectively (Figure 1a). The results verified the importance of selecting an appropriate cut-off value. With smaller cut-off values, the probability of misclassifying negative pools as positive is high. With higher cut-off values, some positive pools will be misclassified. Both will lower the speed and accuracy of decoding. From the results we can infer that the optimal cut-off value is 14; here both the false-negative and false-positive probabilities are very low (Figure 1a).In most studies, because of the rarity of positive samples, the number of positive and negative pools is unequal. Therefore, selecting a cut-off value based on the expected number of errors in classifying pools is a more practical approach. For instance, when there are 30 negative and 10 positive pools mentioned above, the optimal cut-off value is 16 (Figure 1b). In the following simulation experiments, we adopted this kind of scheme unless otherwise stated.

As mentioned, the variance/mean ratio *r* is related to the sequencing platforms and genomes. Because the observed variation is significantly greater than the mean of the sequencing depth, *r* is larger than 1. Different values for *r* yield distinct distributions. The pooling design mentioned above consisting of 30 negative and 10 positive pools was used to estimate the effect of *r* on our methods. We calculated the least depth required for each pool to make the expected number of errors in classifying pools smaller than 1 by increasing the depth gradually (see Additional file 1: Figure S1). From the results, we can see that our method performed even better for smaller *r*, which required less data throughput.

### Performance of the pipeline

To evaluate the performance of our method, we conducted substantial simulations to identify four heterozygous variant carriers among 100 haploid samples. 1000 replicates were done for each pair of design parameters: pool number *t* and column weight *k*. The pooling matrix was designed by collecting random binary vectors with length *t* and weight *k*, meaning that each sample was mixed in *k* of *t* pools. Identical vectors were deleted and the steps were repeated until 100 vectors were obtained to form the matrix.

We used the random function in Perl to simulate the number of reads with and without variants in pooled sequencing. Sequencing error and mixing bias were added to the simulation procedure to bring it closer to a real situation. Sequencing error follows a binomial distribution in sequencing results, and in the simulation the average sequencing error rate was set as 0.01. Mixing bias is caused by the practical difficulty of mixing exactly equal amounts of DNA samples. Based on the study conducted by Shental et al. [13], a random variable following the Gaussian distribution was added to each non-zero element of the pooling matrix to simulate mixing bias. The standard deviation of the Gaussian distribution was 0.05, reflecting up to 5% average noise in the mixed quantities of each sample.

**Figure 2:**
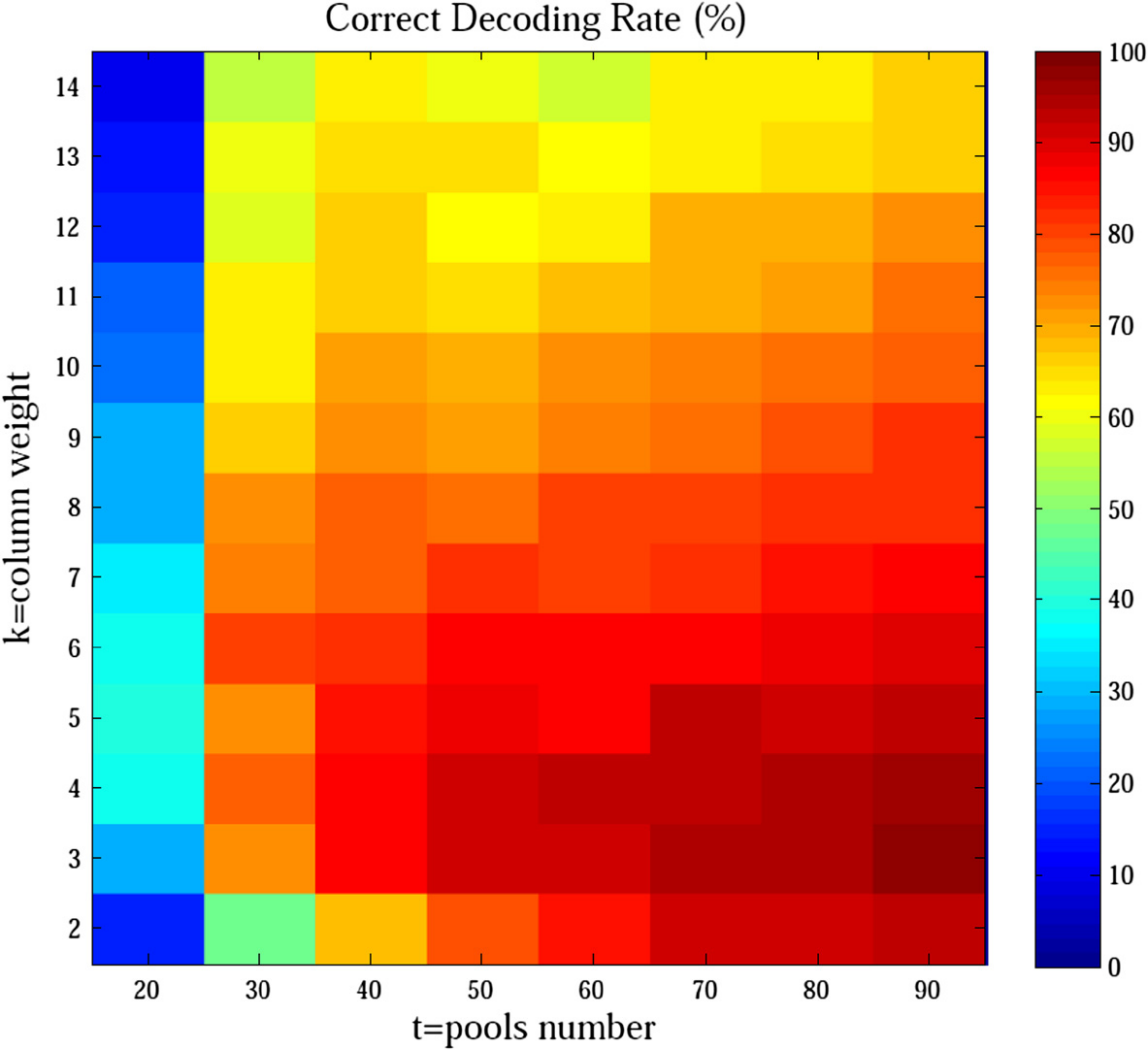
**Correct decoding rates for different column weights.** Correct decoding rates represent the percentage of simulations that identify all the variant carriers correctly.

After simulating the pooled sequencing results containing the sequencing errors and mixing bias, the genotypes of the 100 samples were reconstructed using our decoding algorithm. The correct decoding rates, namely the percentages of simulations that identified all the variant carriers correctly, were determined (Figure 2). The results showed that either a too large or too small *k* negatively influenced the correct decoding rates. Moreover, a large *k* meant more pooling procedures and increased experimental costs. Therefore, a proper column weight *k* is critical for conducting experiments successfully and efficiently.

### Cost-effective overlapping pool sequencing

The column weight *k* denotes the mixing times for each sample in a random *k*-set pool design. For a given number of pools, a *k* that is too large or too small will lower the decoding accuracy. We designed an indicative probability *PI*, which reflects the performance of random *k*-set designs that could be used to choose the optimal column weight *k*.

**Figure 3:**
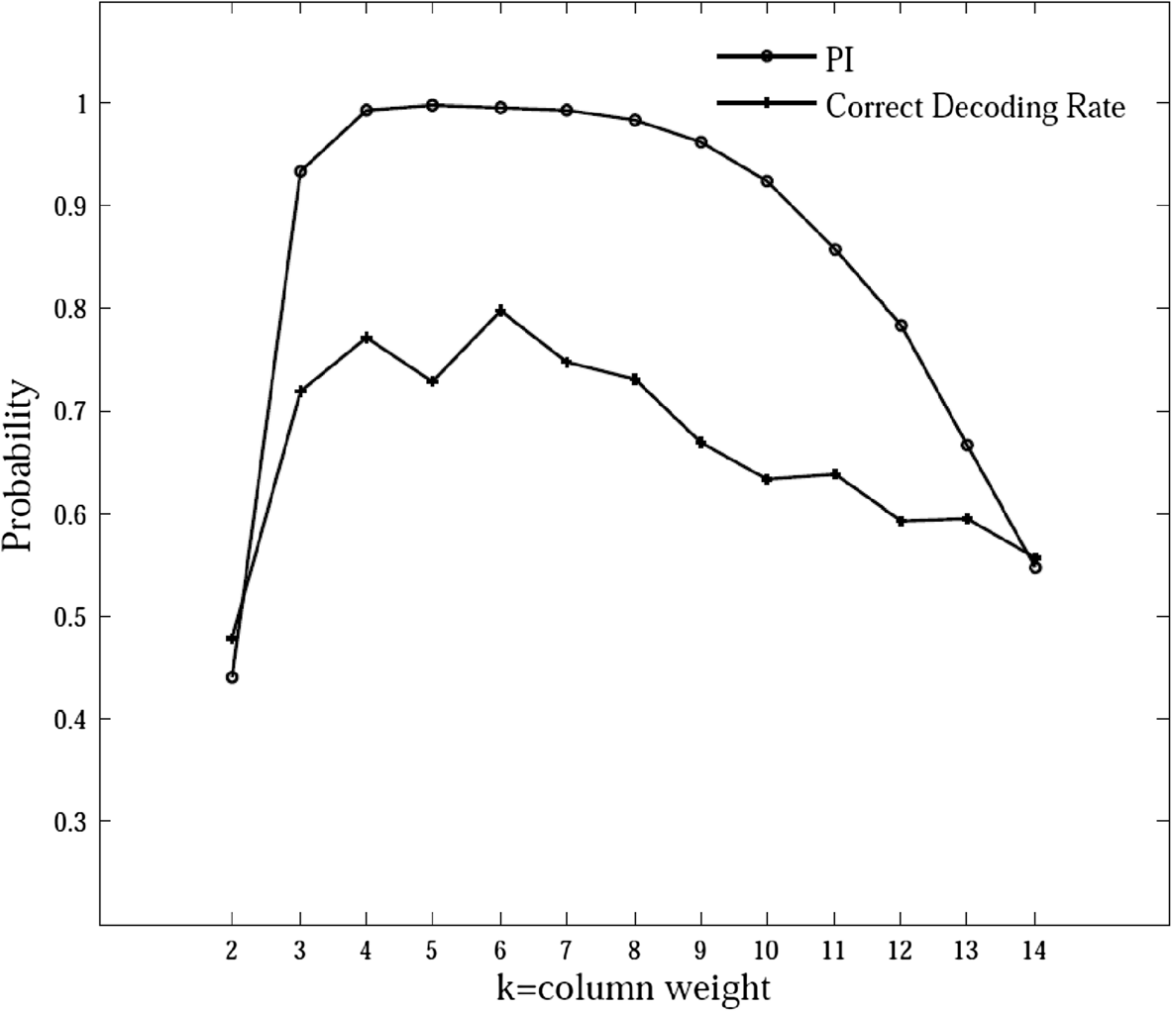
**Correlation between the *PI *value and the correct decoding rate.** Thirty pools were used to identify four heterozygous variant carriers among 100 diploid samples with a depth of 60× for each sample for pooled sequencing. The range of the column weight *k* was 2–14.

We calculated the correct decoding rates for different *k* under the condition that 30 pools were allowed to identify four heterozygous variant carriers among 100 diploid samples by conducting 1000 replicates for each *k*. Next, *PI* was computed based on Eq. (5) and the results are shown in Figure 3. A strong correlation was observed between *PI* and the correct decoding rate (Pearson correlation coefficient = 0.92, *p*-value = 9.8e-06), especially before the correct decoding rate reached the saturation point (*k* = 6, Pearson correlation coefficient = 0.98, *p*-value = 3.4e-3). The *PI* values and correct decoding rates for identifying variant carriers were also obtained under different scenarios (see Additional file 1: Figure S2). All the scenarios showed strong correlations between *PI* and the correct decoding rate before the correct decoding rate reached the saturation point.

For a given pool number *t*, we defined the optimal *k* as the minimum that obtains the maximum *PI* value, which could maximize the correct decoding rate. Designs with optimal *k* require fewer pools or lower sequencing depth. In practice, the optimal *k* is selected by calculating the *PI* value without the need to conduct simulations, thereby greatly reducing the computational time required.

**Figure 4:**
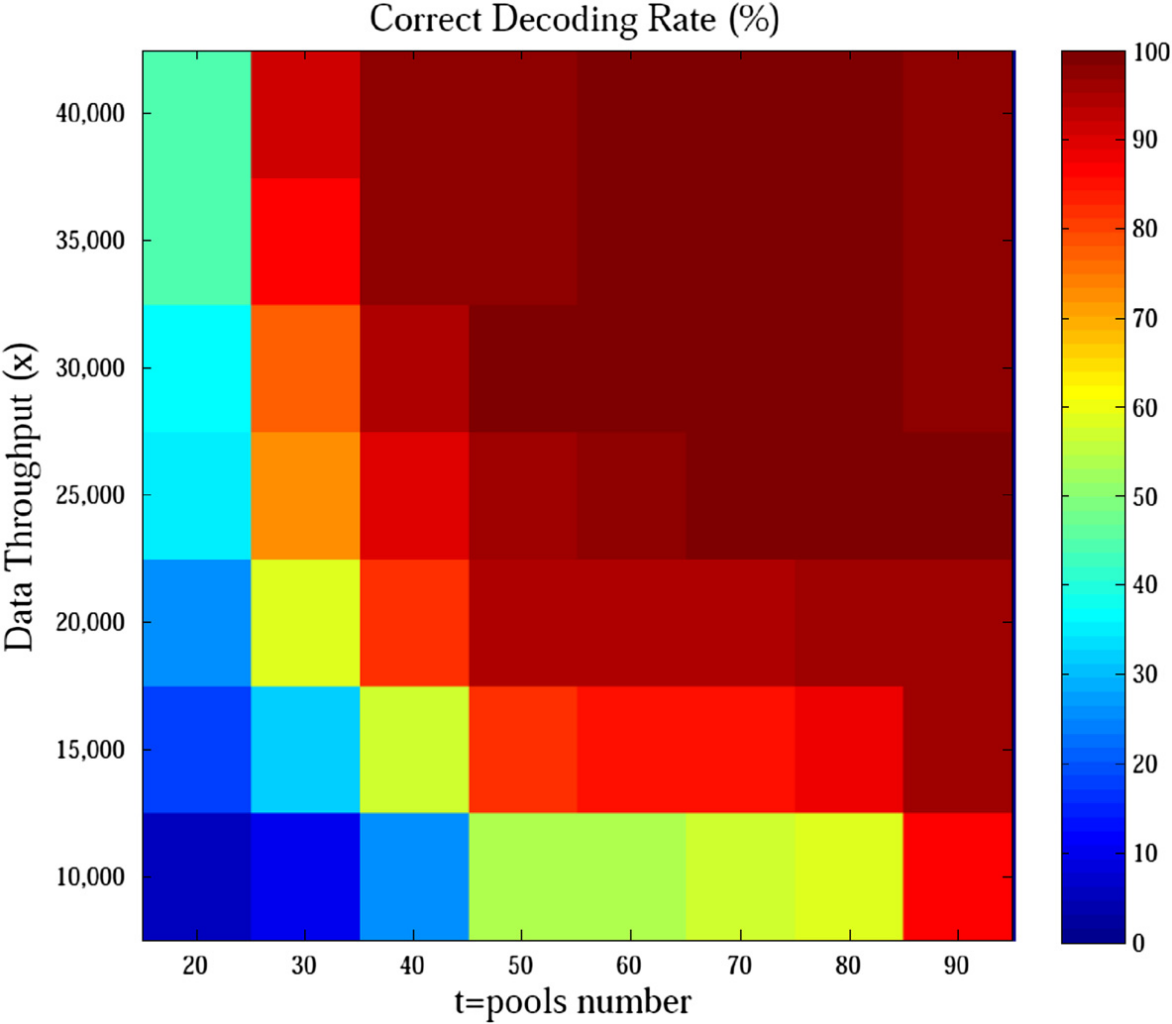
**Performance of overlapping pool sequencing using random *k *-set pool design.** Column weight for each scenario was set to the optimal value to identify four variant carriers among 100 samples.

Next, we conducted a series of simulated overlapping pool sequencing experiments with 20–90 pools and 10,000–40,000× overall sequencing data throughput (Figure 4). One thousand replicates were conducted for each scenario, and the column weight was set as the optimal value (see Additional file 1: Table S1). The correct decoding rates were low when few pools or data throughput were used. However, adequate pools and data throughput achieved higher accuracy but increased the cost, which conflicted with our motivation in this study. There is a trade-off between the number of pools and data throughput. Hence, numerous simulations need to be performed to verify whether a pool number and data throughput pair can succeed in achieving high accuracy (e.g., 95%). Clearly, the optimal design parameters should be selected based on the whole cost of the sequencing experiment.

For a given population with 100 diploid individuals containing one heterozygous variant carrier, we generated several candidate designs in which over 95% of the simulations correctly identified the variant carrier (Table 1). The sequencing region was set to 30 Mb to fit the human exome sequencing project [22]. The cost of a sequencing experiment includes library construction and data production. Using the cost model from our previous work [14], we inferred that the lowest cost design was design II in Table 1. Compared with sequencing separately, which requires sequencing depths of 24.2× for each sample to obtain correct decoding rates over 95%, our method can save at least 50% of the cost. With the same procedure, we generated the most cost-effective designs for variants with different frequencies and different sequencing region sizes (Table 2). For smaller sequencing regions and variants with lower frequencies, there are greater cost reductions with our method compared with those for larger regions and variants with higher frequencies.

**Table 1:** Five candidate designs to identify one heterozygous variant carrier among 100 individuals

**ID of candidate design**	**# of pools**	**Data throughput (Gb)**^**a**^	**Cost**
I	10	567.0	$35,051.0
II	20	292.8	$25,518.4
III	30	268.8	$29,246.4
IV	40	272.4	$34,437.2
V	50	265.2	$39,055.6
Sequencing separately^b^	100	72.6	$53,847.8

^a^Average value from five simulations. Gb is short for gigabases. Data throughput is the sequencing depth multiplied by the length of the sequencing region. ^b^Sequencing separately is the strategy when each sample is sequenced independently. All the candidate designs can identify the variant carrier correctly in 95% of the simulations. The costs were estimated at $500 for one library preparation and $5300 for 100 Gb of data.

**Table 2:** Most cost-effective designs for different scenarios

	**Sequencing region (Mb)**	**Sample size**	**Frequency of variant**	**# of pools**	**Data throughput (Gb)**	**Cost saving**
Haploid sample	5	200	0.5%	20	83.4	85.7%
	5	200	1%	30	124.8	78.6%
	5	200	1.5%	40	128.8	73.4%
Diploid sample	30	200	0.25%	30	669.6	53.4%
	30	100	0.5%	20	292.8	52.6%
	30	100	1%	30	534.6	20.3%

The sequencing region for haploid samples was set as 5 Mb to fit the average length of the bacterial genome. The sequencing region for diploid samples was set as 30 Mb to fit the human exome sequencing.

### Comparisons with current methods

In 2009, benefiting from the Chinese remainder theorem, Erlich et al. [11] put forward the DNA Sudoku design for overlapping pool sequencing. A pattern consistency decoding algorithm was also developed by Erlich et al. [11] to identify variant carriers with the DNA Sudoku design. In 2010, Shental et al. [13] developed a method called compressed sequencing to identify rare variants and their carriers by borrowing techniques from compressed sensing. Two designs were proposed in compressed sequencing: one used pools with a random half of the samples and the other used pools with sizes equal to the square root of the number of samples. We compared the performance of our method in identifying rare variant carriers with the performances of these two methods.

To identify variant carriers in 100 diploid samples, the DNA Sudoku design with parameter *d*_*0*_ = 2 was employed that required 36 pools. To maintain consistency, only 36 pools were allowed for the random *k*-set pool design and compressed sequencing. Since the expected number of positive and negative pools was not clear for the DNA Sudoku design, the cut-off value for the number of reads containing variants to declare a pool to be positive was set based on the false-negative and false-positive rates, and not on the expected number of errors in the classifying pools.

**Figure 5:**
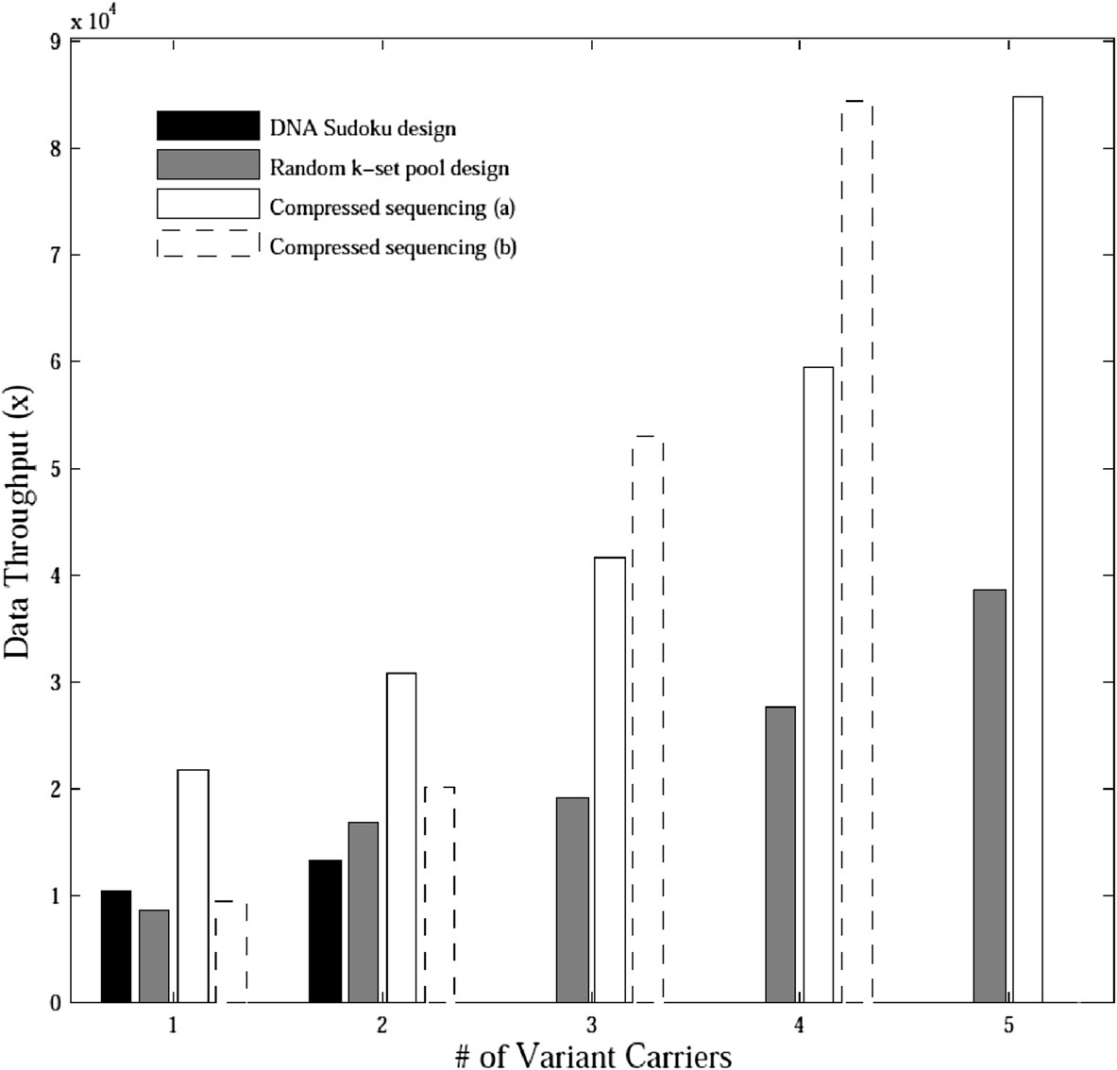
**Least sequencing data throughput required to achieve a 95% correct decoding rate.** Only 36 pools were allowed to identify heterozygous variant carriers among 100 diploid samples. ‘Compressed sequencing (a)’ used pools with a random half of the samples, and ‘compressed sequencing (b)’ used pools with sizes equal to the square root of the number of samples.

With 36 pools, we computed the least sequencing data throughput required for all the methods by increasing the depth gradually, until 95% of the simulations identified all the carriers correctly for various percentages of heterozygous variant carriers (Figure 5, Additional file 1: Table S2). Our method performed better than both the designs in compressed sequencing. The advantages of our method were significant with large numbers of variant carriers. The performance of the DNA Sudoku design was similar to our method when the number of variant carriers was no more than two, but it did not perform well for variants with higher frequencies because of the limited efficiency of the pattern consistency decoding algorithm. For these cases, more pools are required for the DNA Sudoku design than for both our method and compressed sequencing.

**Figure 6:**
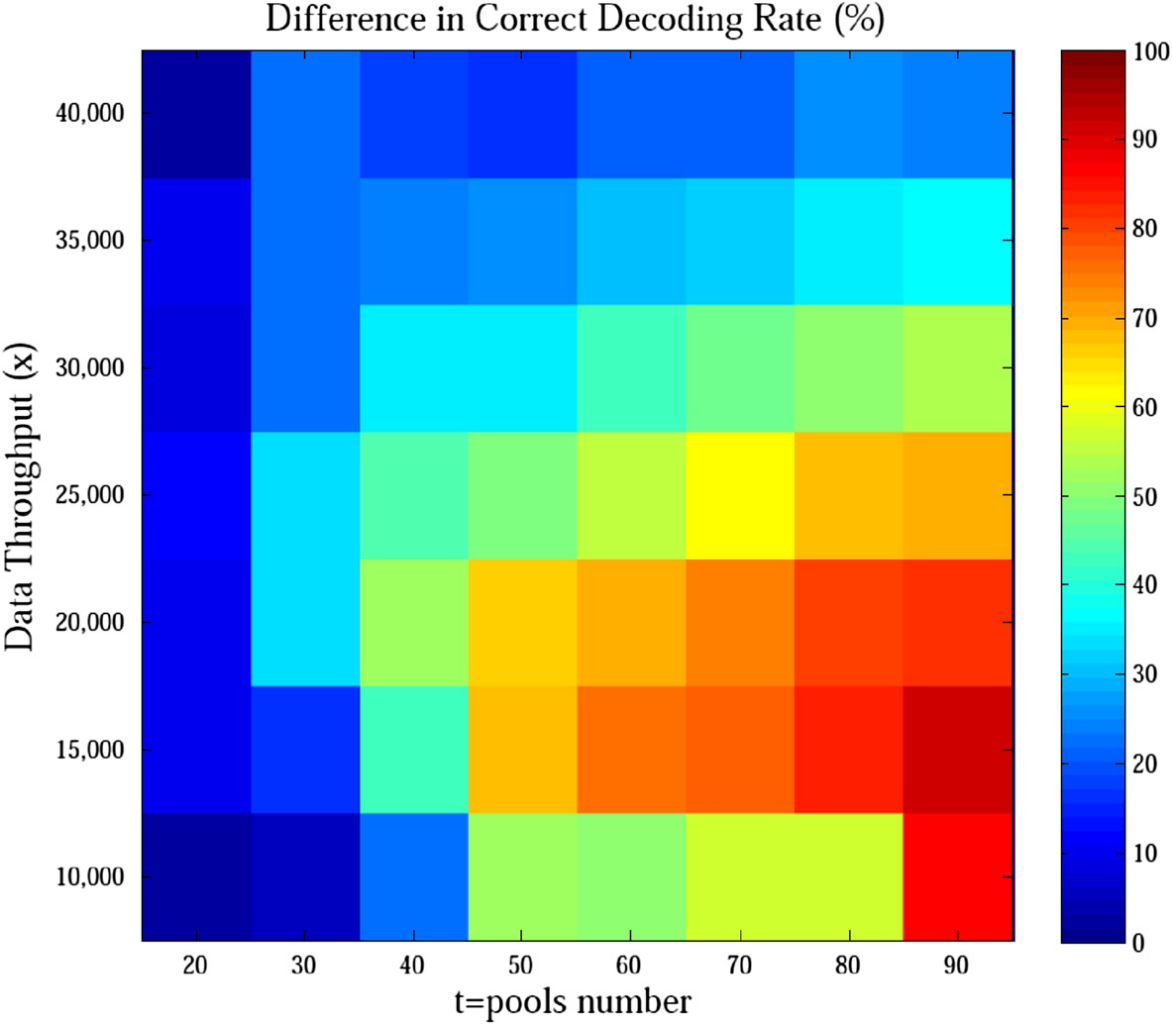
**Difference in correct decoding rate between our method and compressed sequencing.** The design that harnessed pools with a random half of the samples was used for compressed sequencing. The heat map indicates the correct decoding rates using our method minus that of compressed sequencing. Our method performed much better than compressed sequencing, especially when the data throughput was limited.

The DNA Sudoku design is hard to specify for any number of pools. Therefore, we compared only the performance of compressed sequencing with that of our method to identify four heterozygous variant carriers among 100 diploid samples by using the same amounts of pools and sequencing throughput (Figure 6, Additional file 1: Figure S3). Our method performed better for most scenarios, especially when the sequencing throughput was limited.

### Simulation experiment

We applied our method to identify variant carriers among 200 simulated *E. coli* strains. Illumina sequencing reads of two *E. coli* strains were downloaded from GenBank’s Short Read Archive (O157:H7 strain [SRA: ERR018562]) and BGI’s FTP site (O104:H4 strain, ftp://ftp.genomics.org.cn/pub/Ecoli_TY-2482). We treated the O157:H7 strain as the variant carrier and the O104:H4 strain as the normal sample. Bowtie0.12.9 [23] was used to map the O157:H7 reads to the O104:H4 genome, and SAMtools 0.1.19 [24] was used to call single base mutations. Because the mean depth was 134× for O157:H7, mutations with depths lower than 130 or higher than 140 were removed to control the quality; the remaining 1271 mutations were used in the analysis.

We conducted three simulation experiments to validate the ability of our method to identify carriers of variants with frequencies ranging from 0.5 to 1.5%. Based on the results in Table 2, we designed the pooling matrix and sequencing depth so that 95% of the simulations correctly identified the variant carriers. Next, pooled sequencing was conducted by selecting reads randomly from the data set and mixing them *in silico*. Considering up to 5% average noise in the DNA quantities of each sample in the pooling procedure, the number of reads for each sample was revised with a random coefficient following a Gaussian distribution to simulate reality. Bowtie was used to map pooled reads, and Perl scripts were used to count the reads with and without variants that were mapped at the loci of variants. After the decoding procedure, variant carriers could be identified correctly for 91.5–97.9% variants. This result was consistent with the design capability (Table 3).

**Table 3:** Correct decoding rate of our method in the identification of variant carriers

**Experiment**	**Frequency of variant**	**Variant carriers**	**Correct decoding rate**
1	0.5%	4^th^	97.9%
2	1%	164^th^, 193^rd^	93.5%
3	1.5%	31^st^, 90^th^, 141^st^	91.5%

## Conclusions

Here, an efficient method that harnesses random *k*-set pool designs and massively parallel sequencing technologies to identify rare variant carriers is presented. The parameters of the random *k*-set pool design can be selected appropriately depending on an indicative probability. According to the depth model for pooled sequencing, the optimal cut-off value to separate negative pools from positive pools was designed. Taking advantage of the quantitative information in the sequencing results, a heuristic Bayesian decoding algorithm to identify the variant carriers was developed. Compared with the DNA Sudoku design and compressed sequencing, our method showed potential advantages, especially in decreasing the required data throughput. Finally, we applied our method to identify variant carriers among 200 simulated *E. coli* strains using simulated pools and Illumina sequencing data. Our method successfully identified variant carriers at reduced experimental costs.

For the accurate identification of variant carriers, the sequencing depth and pool number must be adequate to overcome sequencing errors and mixing bias. Considering the trade-off between the pool number and data throughput, substantial simulations need to be performed to verify whether a design is capable of identifying all the variant carriers correctly. Because the overall cost of overlapping pool sequencing stems from the pooling procedure, library construction, and data production, the optimal design depends on the whole cost.

Our decoding algorithm identifies the variant carriers by maximizing the posterior probability, and does not depend too much on the rarity of variants. Therefore, our approach can succeed even for low frequency variants. Furthermore, the sequencing qualities that indicate the sequencing error probabilities could be integrated into the calculation of the posterior probability in the decoding procedure to improve the accuracy. Compared with compressed sequencing, our decoding procedure was very time-consuming because of the substantial calculation of the posterior probability. This will be improved in future work.

Further improvement could be made with a reasonable depth model. Although in many studies negative binomial distribution rather than Poisson distribution has been used to fit the sequencing depth, numerous different models exist. We could not determine which model fit the depth distribution best because, in previous studies, these models have not been compared. Additionally, different sequencing procedures and platforms, such as exome sequencing and whole genome sequencing, produce distinct depth distributions. We aim to employ a better depth model to improve the performance of our method.

Our method has the advantage over compressed sequencing because required data throughput is reduced. However, because each sample is sequenced multiple times, the required data throughput is still substantial. Third-generation sequencing technologies [25,26], which significantly reduce the cost for data production, may help to overcome this drawback. We expect that our method could be applied not only in sequencing experiments but also in other fields as long as the pooled experimental results contain quantitative information about the number of positive samples.

## Appendix 1: Derivations of Eq. (5) and Eqs. (7)–(10)

Eq. (5): The indicative probability *PI* is the probability that positive pools are more than the sum of unresolved negative samples and real positive samples. If, *N*_*p*_ is the number of positive pools, $\bar{N}$ is the number of unresolved negative samples, and *d* is the number of positive samples, then *PI* can be written as A(1).

(A1) $$\mathrm{PI}=\sum_{i=p\_\min}^{p\_\max}P\left(N_{p}=i\right)P\left(\bar{N}+d\leq i\right)$$

where *p_min* and *p_max* are the minimum and maximum number of positive pools, respectively.

Because *N*_*p*_=*i* indicates that there are *t* - *i* negative pools, *P*(*N*_*p*_=*i*) can be formulated as A(2). Because $P\left(\bar{N}+d\leq i\right)$ = $P\left(\bar{N}\leq i-d\right)$, $P\left(\bar{N}+d\leq i\right)$ can be formulated as A(3). After integrating A(1)–A(3), *PI* can be formulated as A(4).

(A2) $$P\left(N_{p}=i\right)=\left(\begin{array}{c} t\\ t-i \end{array}\right)\mathrm{NEG}\left(t-i\right)$$

(A3) $$P\left(\bar{N}+d\leq i\right)=\sum_{j=0}^{i-d}P\left(\bar{N}=j\right)$$

(A4) $$\mathrm{PI}=\sum_{i=p\_\min}^{p\_\max}\left(\begin{array}{c} t\\ t-i \end{array}\right)\mathrm{NEG}\left(t-i\right)\sum_{j=0}^{i-d}P\left(\bar{N}=j\right)$$

Eq. (7) and Eq. (9): These equations define the probabilities that *N*_*v*_ reads containing variants are observed in a negative pool (*P*_*nv*_(*N*_*v*_)), and *N*_*n*_ reads without variants are observed in a negative pool (*P*_*nn*_(*N*_*n*_)), respectively. Briefly, *P*_*nv*_(*N*_*v*_) can be written as A(5).

(A5) $$P_{\mathrm{nv}}\left(N_{v}\right)=\sum_{i=N_{v}}^{\infty }P\left(i\right)P_{e}\left(N_{v}|i\right)$$

where *P*(*i*) is the probability that *i* reads are obtained, and *P*_*e*_(*Nv*|*i*) is the probability that *N*_*v*_ errors occur among these *i* reads. Because the depth follows a negative binomial distribution and sequencing errors follow a binomial distribution, these two probabilities can be formulated as A(6) and A(7). In A(6), *D* and *r* are the mean depth of coverage for pooled sequencing and the variance/mean ratio, respectively. In A(7), *p*_*error*_ is the mean sequencing error rate.

(A6) $$P\left(i\right)=\mathrm{NB}\left(i;\frac{D}{r-1},\frac{1}{r}\right)$$

(A7) $$P_{e}\left(N_{v}|i\right)=\mathrm{Bin}\left(N_{v};i,p_{\mathrm{error}}\right)$$

After integrating A(5)–A(7), *P*_*nv*_(*N*_*v*_) can be formulated as A(8).

(A8) $$P_{\mathrm{nv}}\left(N_{v}\right)=\sum_{i=N_{v}}^{\infty }\mathrm{NB}\left(i;\frac{D}{r-1},\frac{1}{r}\right)\mathrm{Bin}\left(N_{v};i,p_{\mathrm{error}}\right)$$

The derivation of the formula for *P*_*nn*_(*N*_*n*_) (A(9)) is similar to the derivation for *P*_*nv*_(*N*_*v*_).

(A9) $$P_{\mathrm{nn}}\left(N_{n}\right)=\sum_{i=N_{n}}^{\infty }\mathrm{NB}\left(i;\frac{D}{r-1},\frac{1}{r}\right)\mathrm{Bin}\left(i-N_{n};i,p_{\mathrm{error}}\right)$$

Eq. (8) and Eq. (10): These equations define the probability that *N*_*v*_ reads containing variants are observed in a positive pool (*P*_*pv*_(*N*_*v*_)) and *N*_*n*_ reads without variants are observed in a positive pool (*P*_*pn*_(*N*_*n*_)), respectively. The observations of a variant in a positive pool consist of two parts: real variants from variant chromosomes, and false variants resulting from sequencing errors. Briefly, *P*_*pv*_(*N*_*v*_) can be written as A(10) where *P*_*N*_(*x*) stands for the probability that *x* reads containing variants stemming from the sequencing results of normal chromosomes, and *P*_*P*_(*O* - *x*) denotes the probability that *O* - *x* reads contain variants from variant chromosomes.

(A10) $$P_{\mathrm{pv}}\left(N_{v}\right)=\sum_{x=0}^{N_{v}}P_{N}\left(x\right)P_{P}\left(N_{v}-x\right)$$

By applying a similar procedure to the one used to obtain A(8) and A(9), *P*_*N*_(*x*) and *P*_*P*_(*N*_*v*_ - *x*) can be formulated as A(11) and A(12). The only difference is the mean sequencing depth of coverage. Because the percentages of variant chromosomes and normal chromosomes are *p* and 1 - *p*, respectively, the mean depths of coverage for sequencing variant chromosomes and normal chromosomes are *pD* and (1 - *p*)*D*, respectively.

(A11) $$P_{n}\left(x\right)=\sum_{i=x}^{\infty }\mathrm{NB}(i;\frac{\left(1-p\right)D}{r-1},\frac{1}{r})\mathrm{Bin}\left(x;i,p_{\mathrm{error}}\right)$$

(A12) $$P_{P}\left(O-x\right)=\sum_{j=O-x}^{\infty }\mathrm{NB}(j;\frac{\mathrm{pD}}{r-1},\frac{1}{r})\mathrm{Bin}\left(j-O+x;j,p_{\mathrm{error}}\right)$$

In the same way, *P*_*pv*_(*N*_*v*_) can be obtained by integrating A(10)–A(12), which is shown as A(13).

(A13) $$P_{\mathrm{pv}}\left(N_{v}\right)=\sum_{x=0}^{N_{v}}\left\{\begin{array}{c} \sum_{i=x}^{\infty }\mathrm{NB}(i;\frac{\left(1-p\right)D}{r-1},\frac{1}{r})\mathrm{Bin}\left(x;i,p_{\mathrm{error}}\right)\times \\ \sum_{j=N_{v}-x}^{\infty }\mathrm{NB}(j;\frac{\mathrm{pD}}{r-1},\frac{1}{r})\mathrm{Bin}\left(j-N_{v}+x;j,p_{\mathrm{error}}\right) \end{array}\right\}$$

Similarly, *P*_*pn*_(*N*_*n*_) can be obtained as shown in A(14).

(A14) $$P_{\mathrm{pn}}\left(N_{n}\right)=\sum_{x=0}^{N_{n}}\left\{\begin{array}{c} \sum_{i=x}^{\infty }\mathrm{NB}(i;\frac{\left(1-p\right)D}{r-1},\frac{1}{r})\mathrm{Bin}\left(i-x;i,p_{\mathrm{error}}\right)\times \\ \sum_{j=N_{n}-x}^{\infty }\mathrm{NB}(j;\frac{\mathrm{pD}}{r-1},\frac{1}{r})\mathrm{Bin}\left(N_{n}-x;j,p_{\mathrm{error}}\right) \end{array}\right\}$$

## Competing interests

The authors declare that they have no competing interests.

## Authors’ contributions

CCC, CL, and XS developed the method. CCC performed the experiments and wrote the manuscript. XS revised the manuscript. All authors read and approved the final manuscript.

## Supplementary Material

Additional file 1: Figure S1 Least depth to make the expected number of errors in classifying pools smaller than 1. **Figure S2.** Correlation between the *PI* value and the correct decoding rate for different scenarios. **Figure S3.** Comparison of the correct decoding rate between our method and compressed sequencing using the second kind of design. **Table S1.** Optimal column weight for various numbers of pools to identify four variant carriers among 100 samples. **Table S2.** Least data throughput required to achieve a 95% correct decoding rate in the identification of heterozygous variant carriers among 100 diploid samples under the condition that only 36 pools are allowed. Click here for file

## References

[B1] Bodmer W Bonilla C Common and rare variants in multifactorial susceptibility to common diseases Nat Genet 2008 40 6 695 701 10.1038/ng.f.136 18509313 PMC2527050

[B2] Manolio TA Collins FS Cox NJ Goldstein DB Hindorff LA Hunter DJ McCarthy MI Ramos EM Cardon LR Chakravarti A Finding the missing heritability of complex diseases Nature 2009 461 7265 747 753 10.1038/nature08494 19812666 PMC2831613

[B3] Nelson MR Wegmann D Ehm MG Kessner D Jean PS Verzilli C Shen J Tang Z Bacanu SA Fraser D An abundance of rare functional variants in 202 drug target genes sequenced in 14,002 people Science 2012 337 6090 100 104 10.1126/science.1217876 22604722 PMC4319976

[B4] Tennessen JA Bigham AW O’Connor TD Fu W Kenny EE Gravel S McGee S Do R Liu X Jun G Evolution and functional impact of rare coding variation from deep sequencing of human exomes Science 2012 337 6090 64 69 10.1126/science.1219240 22604720 PMC3708544

[B5] Golan D Erlich Y Rosset S Weighted pooling—practical and cost-effective techniques for pooled high-throughput sequencing Bioinformatics 2012 28 12 i197 i206 10.1093/bioinformatics/bts208 22689761 PMC3371840

[B6] Shendure J Ji H Next-generation DNA sequencing Nat Biotechnol 2008 26 10 1135 1145 10.1038/nbt1486 18846087

[B7] Patterson N Gabriel S Combinatorics and next-generation sequencing Nat Biotechnol 2009 27 9 826 827 10.1038/nbt0909-826 19741641

[B8] Ding-Zhu D Hwang FK Combinatorial group testing and its applications 2000 APPLIED MATHEMATICS: SERIES ON 12

[B9] Candes EJ Romberg JK Tao T Stable signal recovery from incomplete and inaccurate measurements Commun Pure Appl Math 2006 59 8 1207 1223 10.1002/cpa.20124

[B10] Donoho DL Compressed sensing IEEE Trans Inf Theory 2006 52 4 1289 1306

[B11] Erlich Y Chang K Gordon A Ronen R Navon O Rooks M Hannon GJ DNA Sudoku—harnessing high-throughput sequencing for multiplexed specimen analysis Genome Res 2009 19 7 1243 1253 10.1101/gr.092957.109 19447965 PMC2704425

[B12] Prabhu S Pe’Er I Overlapping pools for high-throughput targeted resequencing Genome Res 2009 19 7 1254 1261 10.1101/gr.088559.108 19447964 PMC2704440

[B13] Shental N Amir A Zuk O Identification of rare alleles and their carriers using compressed se (que) nsing Nucleic Acids Res 2010 38 19 e179 e179 10.1093/nar/gkq675 20699269 PMC2965256

[B14] Cao C-C Li C Huang Z Ma X Sun X Identifying rare variants with optimal depth of coverage and cost-effective overlapping pool sequencing Genet Epidemiol 2013 37 820 830 10.1002/gepi.21769 24166758

[B15] Bruno WJ Knill E Balding DJ Bruce D Doggett N Sawhill W Stallings R Whittaker CC Torney DC Efficient pooling designs for library screening Genomics 1995 26 1 21 30 10.1016/0888-7543(95)80078-Z 7782082

[B16] Ngo HQ Du DZ A survey on combinatorial group testing algorithms with applications to DNA library screening Discrete mathematical problems with medical applications 2000 55 171 182

[B17] Hwang F Random k-set pool designs with distinct columns Probability in the Engineering and Informational Sciences 2000 14 1 49 56

[B18] Barillot E Lacroix B Cohen D Theoretical analysis of library screening using a N-dimensional pooling strategy Nucleic Acids Res 1991 19 22 6241 6247 10.1093/nar/19.22.6241 1956784 PMC329134

[B19] Sarin S Prabhu S O’Meara MM Pe’er I Hobert O Caenorhabditis elegans mutant allele identification by whole-genome sequencing Nat Methods 2008 5 10 865 10.1038/nmeth.1249 18677319 PMC2574580

[B20] Anders S Huber W Differential expression analysis for sequence count data Genome Biol 2010 11 10 R106 10.1186/gb-2010-11-10-r106 20979621 PMC3218662

[B21] Miller CA Hampton O Coarfa C Milosavljevic A ReadDepth: a parallel R package for detecting copy number alterations from short sequencing reads PLoS One 2011 6 1 e16327 10.1371/journal.pone.0016327 21305028 PMC3031566

[B22] Ng SB Turner EH Robertson PD Flygare SD Bigham AW Lee C Shaffer T Wong M Bhattacharjee A Eichler EE Targeted capture and massively parallel sequencing of 12 human exomes Nature 2009 461 7261 272 276 10.1038/nature08250 19684571 PMC2844771

[B23] Langmead B Trapnell C Pop M Salzberg SL Ultrafast and memory-efficient alignment of short DNA sequences to the human genome Genome Biol 2009 10 3 R25 10.1186/gb-2009-10-3-r25 19261174 PMC2690996

[B24] Li H Handsaker B Wysoker A Fennell T Ruan J Homer N Marth G Abecasis G Durbin R The sequence alignment/map format and SAMtools Bioinformatics 2009 25 16 2078 2079 10.1093/bioinformatics/btp352 19505943 PMC2723002

[B25] Clarke J Wu HC Jayasinghe L Patel A Reid S Bayley H Continuous base identification for single-molecule nanopore DNA sequencing Nat Nanotechnol 2009 4 4 265 270 10.1038/nnano.2009.12 19350039

[B26] Eid J Fehr A Gray J Luong K Lyle J Otto G Peluso P Rank D Baybayan P Bettman B Real-time DNA sequencing from single polymerase molecules Science 2009 323 5910 133 138 10.1126/science.1162986 19023044

