# Supplementary material for: Quantitative group testing-based overlapping pool sequencing to identify rare variant carriers
Source: BMC Bioinformatics. 2014 Jun 17;15:195. doi: 10.1186/1471-2105-15-195 (PMC4229885; doi:10.1186/1471-2105-15-195)
Supplement: Additional file 1: Figure S1 — Least depth to make the expected number of errors in classifying pools smaller than 1. Figure S2. Correlation between the PI value and the correct decoding rate for different scenarios. Figure S3. Comparison of the correct decoding rate between our method and compressed sequencing using the second kind of design. Table S1. Optimal column weight for various numbers of pools to identify four variant carriers among 100 samples. Table S2. Least data throughput required to achieve a 95% correct decoding rate in the identification of heterozygous variant carriers among 100 diploid samples under the condition that only 36 pools are allowed. [file 1471-2105-15-195-S1.docx]

## Figure S1 Least depth to make the expected number of errors in classifying pools smaller than 1. Poisson distribution is used to fit the sequencing depth when *r* is 1, negative binomial distribution is used for *r* larger than 2.


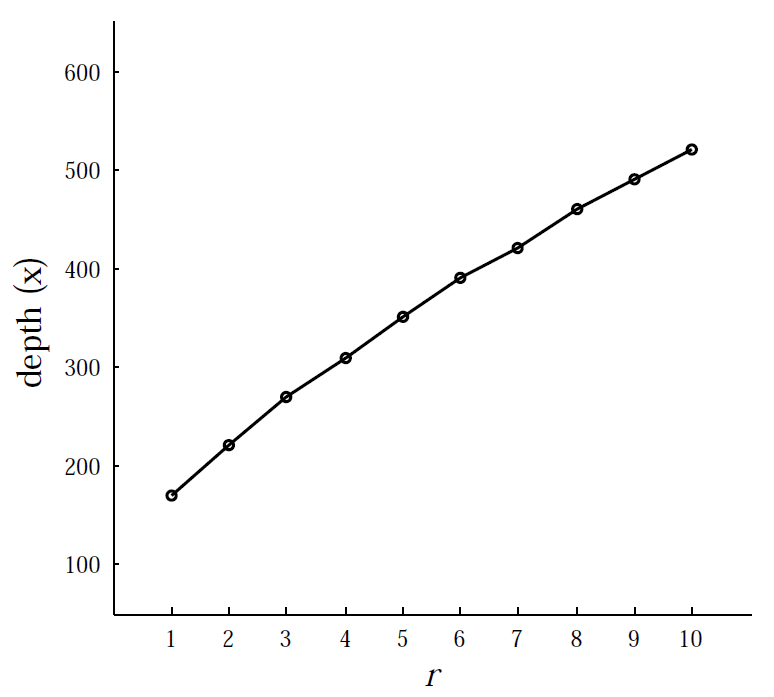


## Figure S2 Correlation between the *PI* value and the correct decoding rate for different scenarios. (a) Identify four heterozygous variant carriers among 100 diploid samples by using 40 pools with a depth of 60× for each sample. Pearson correlation coefficient (Pcc) is 0.99 for *k* smaller than 5, *p*-value = 4.4e-3. (b) Identify four heterozygous variant carriers among 100 diploid samples by using 30 pools with a depth of 70× for each sample. Pcc is 0.99 for *k* smaller than 6, *p*-value = 2.8e-3. (c) Identify four heterozygous variant carriers among 200 diploid samples by using 40 pools with a depth of 60× for each sample. Pcc is 0.99 for *k* smaller than 5, *p*-value = 5.5e-2. (d) Identify four heterozygous variant carriers among 200 diploid samples by using 40 pools with a depth of 70× for each sample. Pcc is 0.99 for *k* smaller than 5, *p*-value = 3.5e-2. All showed strong correlations between the *PI* and the correct decoding rates before the correct decoding rates reached the saturation point.


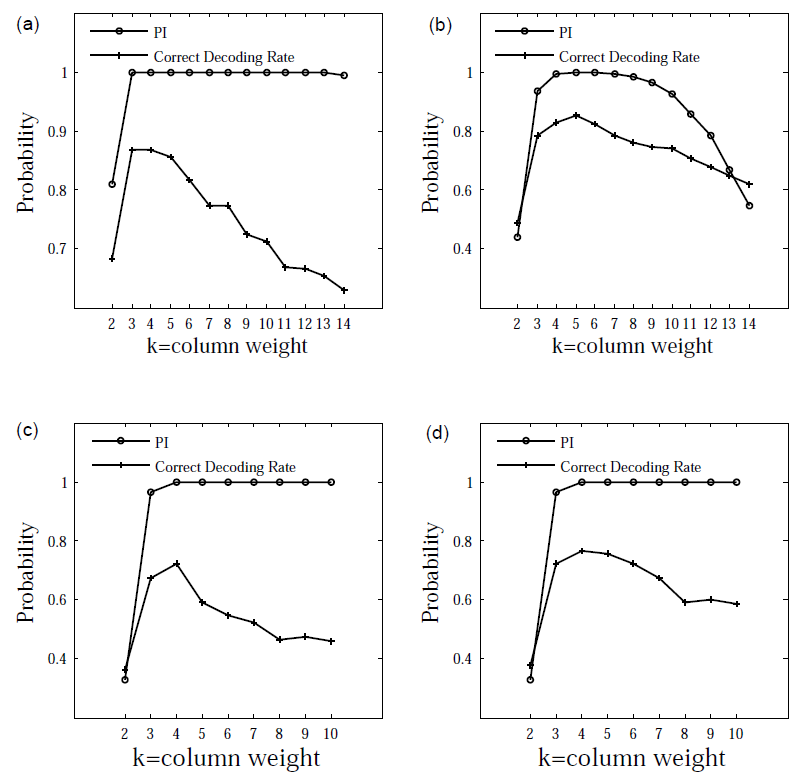


## Figure S3 Comparison of the correct decoding rate between our method and compressed sequencing using the second kind of design. Data in the heat map indicates the correct decoding rates using our method minus that of compressed sequencing.


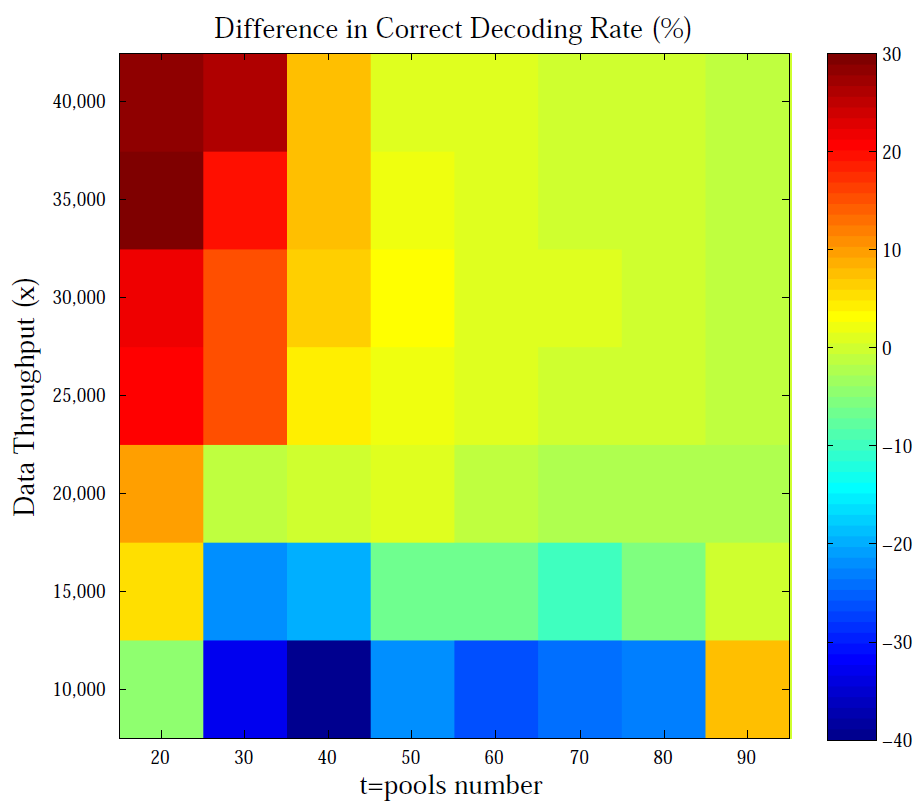


## Table S1. Optimal column weight for various numbers of pools to identify four variant carriers among 100 samples.

| # of pools | Optimal column weight |
| --- | --- |
| 20 | 5 |
| 30 | 5 |
| 40 | 4 |
| 50 | 3 |
| 60 | 3 |
| 70 | 3 |
| 80 | 3 |
| 90 | 2 |

## Table S2. Least data throughput required to achieve a 95% correct decoding rate in the identification of heterozygous variant carriers among 100 diploid samples under the condition that only 36 pools are allowed.

| # of variant carriers | Average # of samples per pool | | | | Depth per sample for pooled sequencing (x) | | | | Data throughput (x) | | | |
| --- | --- | --- | --- | --- | --- | --- | --- | --- | --- | --- | --- | --- |
|  | CS(a) | CS(b) | D | R | CS(a) | CS(b) | D | R | CS(a) | CS(b) | D | R |
| 1 | 50 | 10 | 8.3 | 5.5 | 12 | 26 | 34 | 43 | 21,600 | 9,360 | 10,200 | 8,600 |
| 2 | 50 | 10 | 8.3 | 8.3 | 17 | 56 | 44 | 56 | 30,600 | 20,160 | 13,200 | 16,800 |
| 3 | 50 | 10 | 8.3 | 8.3 | 23 | 147 | N/A | 64 | 41,400 | 52,920 | N/A | 19,200 |
| 4 | 50 | 10 | 8.3 | 11.1 | 33 | 234 | N/A | 69 | 59,400 | 84,240 | N/A | 27,600 |
| 5 | 50 | 10 | 8.3 | 13.8 | 47 | N/A | N/A | 77 | 84,600 | N/A | N/A | 38,500 |

CS(a) denotes compressed sequencing that used pools with a random half of the samples.

CS(b) denotes compressed sequencing that used pools with sizes equal to the square root of the number of samples

D denotes the DNA Sudoku design.

R denotes the random *k*-set pool design.

N/A denotes that no data is available. We stopped the calculation before too much time is consumed.
